# Redox-Responsive Polymersomes as Smart Doxorubicin Delivery Systems

**DOI:** 10.3390/pharmaceutics14081724

**Published:** 2022-08-18

**Authors:** Carmen Ferrero, Marta Casas, Isidoro Caraballo

**Affiliations:** Departamento de Farmacia y Tecnología Farmacéutica, Facultad de Farmacia, Universidad de Sevilla, C/Prof. García González No. 2, 41012 Sevilla, Spain

**Keywords:** polymersome, triblock copolymer mPEG–PDH–mPEG, redox-responsive, doxorubicin hydrochloride, smart drug delivery systems, drug release kinetics

## Abstract

Stimuli-responsive polymersomes have emerged as smart drug delivery systems for programmed release of highly cytotoxic anticancer agents such as doxorubicin hydrochloride (Dox·HCl). Recently, a biodegradable redox-responsive triblock copolymer (mPEG–PDH–mPEG) was synthesized with a central hydrophobic block containing disulfide linkages and two hydrophilic segments of poly(ethylene glycol) methyl ether. Taking advantage of the self-assembly of this amphiphilic copolymer in aqueous solution, in the present investigation we introduce a solvent-exchange method that simultaneously achieves polymersome formation and drug loading in phosphate buffer saline (10 mM, pH 7.4). Blank and drug-loaded polymersomes (5 and 10 wt.% feeding ratios) were prepared and characterized for morphology, particle size, surface charge, encapsulation efficiency and drug release behavior. Spherical vesicles of uniform size (120–190 nm) and negative zeta potentials were obtained. Dox·HCl was encapsulated into polymersomes with a remarkably high efficiency (up to 98 wt.%). In vitro drug release studies demonstrated a prolonged and diffusion-driven release at physiological conditions (~34% after 48 h). Cleavage of the disulfide bonds in the presence of 50 mM glutathione (GSH) enhanced drug release (~77%) due to the contribution of the erosion mechanism. Therefore, the designed polymersomes are promising candidates for selective drug release in the reductive environment of cancer cells.

## 1. Introduction

Doxorubicin (Dox) is a broad-spectrum antibiotic used as a first line treatment against various solid tumors. However, due to its very narrow therapeutic index, direct administration of Dox is frequently associated with severe side effects such as cumulative cardiotoxicity and nephrotoxicity. These major drawbacks can be overcome by encapsulating Dox in nanocarriers like polymersomes, that avoid non-specific drug distribution, reducing the acute toxicity of the free drug and improving its therapeutic efficacy [1,2,3,4,5].

Polymersomes are self-assembled vesicles prepared from synthetic, biodegradable and biocompatible amphiphilic block copolymers, which have attracted growing interest in recent years for their wide use as drug delivery systems. Polymersomes contain an aqueous core suitable for encapsulating hydrophilic drugs surrounded by a hydrophobic bilayer membrane that can load hydrophobic molecules. The size, membrane thickness, mechanical strength and permeability of polymersomes can be modulated by changing the copolymer composition and molecular weight. This versatility is a clear advantage over nanocarriers of similar architecture such as liposomes because adjusting lipid properties is limited. Other benefits of polymersomes over their lipidic counterparts are in better colloidal stability, higher mechanical robustness, tunable functionalization, higher drug-loading capacity, improved controlled release properties and prolonged circulation time [5,6,7,8,9,10,11].

Due to the leaky vasculature in tumor tissues, polymersomes (≤200 nm) can passively accumulate within a tumor cell through enhanced permeability and retention (EPR). Polymersomes with hydrophilic blocks such as poly(ethylene glycol) (PEG) can avoid plasmatic protein adsorption to prolong blood circulation half-life. The poorly developed lymphatic drainage of pegylated polymersomes also contributes to their accumulation at the tumor site [4,12,13,14].

To achieve active targeted drug delivery and improved therapeutic efficiency and specificity of polymersomes, stimuli-responsive linkages can be incorporated into the design of the constituent copolymer blocks. Stimuli both internal (pH, temperature, redox potential, enzymes) and external (light, temperature, ultrasound, magnetic field) have been used to achieve controlled drug release and improve biodegradation of the polymeric nanosystem at the tumor site [15,16,17,18,19,20,21,22,23,24]. Regarding scalability, costs and regulatory considerations, the application of internal triggers has considerable advantages [25,26]. Moreover, as the cancer cells contain higher levels of reducing agents (glutathione -GSH- concentration 2–50 mM) compared to healthy cells (2–20 µM), redox-responsive polymersomes are a promising smart platform for intracellular drug delivery into a tumor [16,27,28,29,30,31,32].

Based on this concept, the redox-responsive amphiphilic triblock copolymer mPEG–PDH–mPEG was synthesized in a previous study [33] using a simple, reproducible and easily scalable methodology. The hydrophobic central block (PDH) containing multiple disulfide bonds (S–S) was prepared in the first step, which was further extended with poly(ethylene glycol) methyl ether (mPEG). As outlined before, the hydrophilic PEG blocks impart structure stability and increases polymersome circulation time. The hydrophobic PDH block effects the polymersome response to the redox environment as the disulfide bonds are easily reduced to thiol groups (2 SH per S–S linkage) under the hypoxic conditions of the tumor microenvironment. The copolymer was chemically characterized and its ability to self-assemble in water at 37 °C tested. The preformed vesicles were loaded with the model drug by the gradient concentration difference between donor and acceptor compartments separated by a dialysis membrane. However, drug permeation across the dialysis membrane would not ensure the diffusion of drug molecules into the vesicle.

As parameters such as non-solvent pH and temperature could affect the properties of self-assembled structures [34], the aim of the present study was to investigate the dynamic self-assembly of this amphiphilic triblock copolymer in phosphate buffer saline (PBS) (10 mM, pH 7.4) at room temperature. Furthermore, an optimized and reproducible method was established to accomplish polymersome formation and drug loading simultaneously with high encapsulation efficiency. Doxorubicin hydrochloride (Dox·HCl) was chosen as a model hydrophilic chemotherapeutic drug due to its wide use in cancer therapy. The physicochemical characteristics of the polymersomes––size, zeta potential, morphology and drug encapsulation efficiency––were evaluated. In vitro drug release behavior was also analyzed in detail fitting the experimental data to different kinetic models to elucidate the drug release mechanism from the designed polymersomes.

## 2. Materials and Methods

### 2.1. Materials

**Figure 1:**
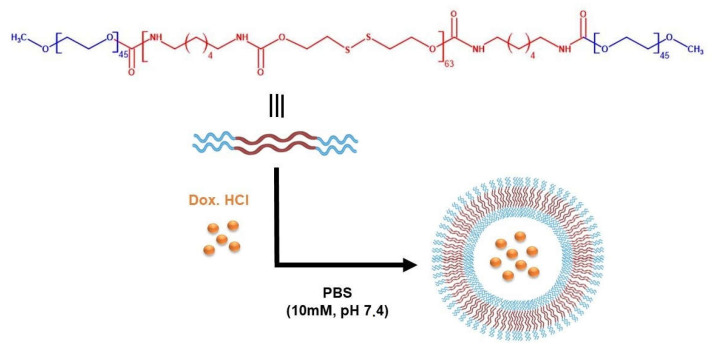
Schematic representation of the molecular structure of mPEG–PDH–mPEG copolymer and the self-assembly of drug-loaded polymersomes.

The reduction-sensitive mPEG–PDH–mPEG triblock copolymer was kindly supplied by Departamento de Química Orgánica y Farmacéutica (Facultad de Farmacia, Universidad de Sevilla) and synthesized as previously reported [33]. Briefly, the hydrophobic disulfide-containing block (PDH) was obtained by the reaction of commercial 2,2′-dithiodiethanol (DiT) and hexamethylene diisocyanate (HMDI) at room temperature, under an inert atmosphere, using N,N-dimethylacetamide (DMA) as a solvent and dibutyltin (II) dilaurate as a catalyst. The mixture was further reacted with a solution of 2000 g·mol^−1^ poly(ethylene glycol) methyl ether (mPEG_2000_) in DMA to afford the triblock copolymer (Figure 1). A white solid with a yield of 82% was obtained, its chemical structure characterized by ^1^H and ^13^C NMR and FTIR spectroscopies. The weight-average molecular weight (M_w_) of this copolymer, as determined by size-exclusion chromatography (SEC), was 25,804 g·mol^−1^, with a narrow polidispersity (M_w_/M_n_ 1.02). The thermal behavior of the copolymer examined by Differential Scanning Calorimetry (DSC) and Thermogravimetric Analysis (TGA) revealed the semicrystalline nature of the material, with a glass transition temperature (T_g_) of 1 °C and a melting temperature (T_m_) of 145 °C. Moreover, the material shows high thermal stability, with a decomposition onset temperature (T_d_) of 259 °C.

Doxorubicin hydrochloride (Dox·HCl) (>98.0% purity, M_w_ 579.98 g·mol^−1^, orange-to- red powder) (batch LC49367), a water-soluble anticancer drug, was purchased from AK Scientific, Inc. (Union City, CA, USA).

L-Glutathione reduced (GSH) (batch 8M014235) (Panreac, Barcelona, Spain) was used as a tripeptide responsible for the reduction of disulfide linkages.

Anhydrous tetrahydrofuran (THF) (batch 0001599876) (Panreac, Barcelona, Spain) and phosphate buffer saline (PBS) (10 mM, pH 7.4) were used as solvent and non-solvent, respectively, for the solvent-switching method.

Ultrapure water (18 MΩ·cm resistivity) was obtained from Milli-Q system (Merck Millipore, Darmstdat, Germany) and used for all studies.

All other solvents and chemical reagents were of either High Performance Liquid Chromatography (HPLC) or analytical grades.

Spectra/Por^®^ 3 (MWCO 3.5 kD) dialysis membranes were supplied by VWR International Eurolab, S.L. (Barcelona, Spain) and used for the drug loading experiments. Dialysis float devices (Spectra/Por^®^ Float-A-Lyzer^®^ G2, MWCO 3.5–5 kD; volume 1 mL) were supplied by Sigma-Aldrich (St. Louis, MI, USA) and used for the drug release experiments.

### 2.2. Methods

#### 2.2.1. Preparation of Blank Polymersomes

The polymersomes were prepared using the solvent-switching or solvent-exchange method as follows. After a preliminary screening of different experimental conditions, a weighed amount of the triblock copolymer (50 mg) was dissolved in 5 mL of anhydrous THF in a round-bottom flask. Then, 10 mL of PBS (10 mM, pH 7.4) was dropwise added to the organic phase at a rate of 0.1 mL·h^−1^ (Syringe Pump 11 Elite, Harvard Apparatus, Holliston, MA, USA) and under vigorous magnetic stirring. Subsequently, the organic solvent was evaporated under exposure to air for 48 h. A cloudy dispersion was obtained with a polymer concentration of 5 mg·mL^−1^.

#### 2.2.2. Morphology, Particle Size and Zeta Potential Measurements

Transmission Electron Microscopy (TEM) measurements were carried out to observe the morphology of the prepared polymersomes. The polymersomes dispersion was dropped onto 200 mesh carbon-coated copper grids––the extra sample wiped off with filter paper–– and air dried. TEM images were captured with a Philips CM200 transmission electron microscope (Philips Electron Optics GmbH, Hamburg, Germany) operating at 200 kV, available at Centro de Investigación, Tecnología e Innovación (CITIUS) (Universidad de Sevilla).

The intensity-average diameter and size distribution of the polymersomes were measured by Dynamic Light Scattering (DLS) using disposable polystyrene cuvettes. Briefly, the polymersomes suspended in PBS (10 mM, pH 7.4) were diluted 20% *v/v* in MilliQ ultrapure water, sonicated for 20 min and filtered (0.45 µm). Samples were then analyzed with a Zetasizer Nano ZS (Malvern Instruments Ltd., Worcestershire, UK) equipped with a 4 mW He–Ne laser operating at 633 nm through back-scattering detection (173°). All measurements were performed in triplicate at 25 °C.

The electrophoretic mobility (µ) of the polymersome dispersion was also measured by the same equipment using disposable folded capillary cells. The electrophoretic mobility was converted to the zeta (ζ) potential using the Smoluchowski approximation. All measurements were made in triplicate at 25 °C.

The particle size and zeta potential were analysed periodically by DLS to evaluate polymersome stability after storage at 4 °C for 1 month.

#### 2.2.3. Preparation and Characterization of Drug-Loaded Polymersomes

For encapsulation of Dox·HCl, the preparation method of blank polymersomes was modified to simultaneously accomplish the self-assembly of mPEG–PDH–mPEG copolymer and drug loading (Figure 1). The triblock copolymer (10 mg) was dissolved in anhydrous THF (5 mL) in a round-bottomed flask. Then, 1 mL of Dox·HCl aqueous solution (concentration adjusted to obtain theoretical 5 wt.% or 10 wt.% drug/polymer feed weight ratios) was dropwise added (0.15 mL·h^−1^) to the organic phase followed by the addition of 9 mL of PBS (10 mM, pH 7.4) (Syringe Pump 11 Elite, Harvard Apparatus, Holliston, MA, USA) under vigorous magnetic stirring. The resulting dispersion was then sealed into a dialysis bag (Spectra/Por^®^ 3, MWCO 3.5 kD) and dialyzed against PBS (1 L) for 6 h (renewed every 2 h) to remove THF and unencapsulated drug if present. The polymersome concentration after dialysis was approximately 0.5 mg·mL^−1^. The whole process was performed in the dark to avoid photodegradation of Dox·HCl.

**Figure 2:**
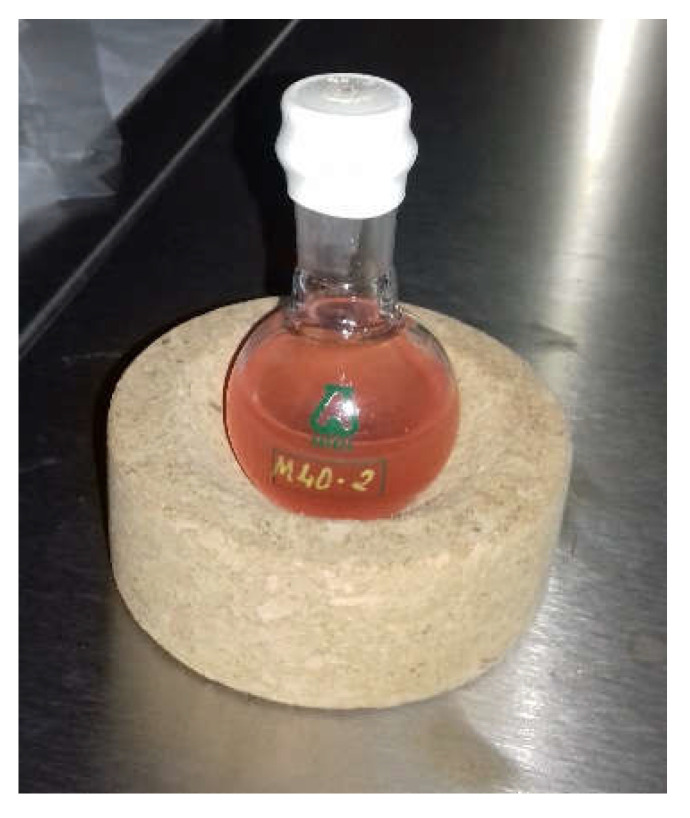
Photograph of the drug-loaded (10 wt.% Dox·HCl) polymersome dispersion.

The polymersome dispersion, with a visible orange opalescence (Figure 2) indicative of drug encapsulation, was wrapped in aluminum foil and stored in a refrigerator at 4 °C.

The morphology of drug-loaded polymersomes was evaluated by conventional TEM following the method for blank polymersomes. In addition, other microscopic techniques available at CITIUS (Universidad de Sevilla) were applied to drug-loaded (10 wt.% Dox·HCl) polymersomes to further investigate the morphology of the self-assembled structures and the drug distribution.

Hence, samples of drug-loaded (10 wt.% Dox·HCl) polymersomes were subjected to negative staining for imaging using a Zeiss Libra 120 transmission electron microscope (Carl Zeiss Microscopy, Oberkochen, Germany) operating at 120 kV. The polymersome dispersion was dropped on a 300-mesh Formvar-coated copper grid and, after 1 min of incubation, the extra sample was wiped off with filter paper. Subsequently, the sample was stained using 1.0 wt.% phosphotungstic acid solution for 1.5 min, the excess dye was blotted using filter paper, and air dried samples were imaged.

The drug distribution within the polymersomes was also verified by Confocal Laser Scanning Microscopy (CLSM) (Zeiss LSM7 Duo, Carl Zeiss Microscopy, Oberkochen, Germany). The dispersion was mounted onto a microscope slide and fixed. The fluorescence of Dox·HCl was examined with excitation wavelength (λ_exc_) at 488 nm and emission wavelength (λ_emiss_) at 596 nm.

The size and zeta potential measurements of drug-loaded polymersomes were performed by DLS following the method for blank polymersomes.

#### 2.2.4. Calculation of Drug Encapsulation Efficiency and Drug Loading Content

Drug encapsulation efficiency (DEE, wt.%) and drug loading content (DLC, wt.%) were determined by High Performance Liquid Chromatography (HPLC) (Agilent 1200, Waldbronn, Germany). Samples (1 mL) of the polymersome dispersion were centrifuged at 10,000 rpm and 4 °C for 15 min. Supernatant and precipitate were separated, and the supernatant diluted with 1 mL DMSO and vortexed for 1 min. Samples were filtered (0.45 µm) prior analysis and eluted through a Inertsil ODS-3 C_18_ column (5 µm, 250 mm × 4.6 mm, GL Sciences Inc., Tokyo, Japan) at 30 °C. The mobile phase consisted of a 49:20:31 mixture (*v*/*v*) of pure HPLC-grade methanol–acetonitrile–phosphate buffer solution (25 mM Na_2_HPO_4_ and 30 mM KH_2_PO_4_ with pH adjusted to 5.0), delivered at a constant flow rate of 1 mL·min^−1^. The injection volume was 30 µL and the detection wavelength was set at 480 nm. The retention time for doxorubicin was 4.7 min. The data were acquired and processed by means of HP ChemStation software. Samples concentration was determined from a calibration curve of peak area vs. concentration of doxorubicin in DMSO in the range of interest (r^2^ = 0.999).

The drug encapsulation efficiency (DEE, wt.%) and drug loading content (DLC, wt.%) were indirectly determined using Equations (1) and (2), respectively:

(1) $$\mathrm{DEE}\left(\mathrm{wt}.\%\right)=\frac{\left(\mathrm{Weight}\;\mathrm{of}\;\mathrm{feeding}\;\mathrm{drug}-\mathrm{Weight}\;\mathrm{of}\;\mathrm{drug}\;\mathrm{in}\;\mathrm{supernatant}\right)}{\mathrm{Weight}\;\mathrm{of}\;\mathrm{feeding}\;\mathrm{drug}}\times 100$$

(2) $$\mathrm{DLC}\left(\mathrm{wt}.\%\right)=\frac{\left(\mathrm{Weight}\;\mathrm{of}\;\mathrm{feeding}\;\mathrm{drug}-\mathrm{Weight}\;\mathrm{of}\;\mathrm{drug}\;\mathrm{in}\;\mathrm{supernatant}\right)}{\mathrm{Weight}\;\mathrm{of}\;\mathrm{block}\;\mathrm{copolymer}}\times 100$$

Moreover, the absence of unencapsulated drug in the dialysis reservoirs was confirmed by HPLC measurements.

#### 2.2.5. In Vitro Drug Release Study

The in vitro release of doxorubicin from the polymersomes was assessed by the dialysis method. Drug-loaded (10 wt.% Dox·HCl) polymersomes dispersion (1 mL) was introduced into a dialysis float device (Spectra/Por^®^ Float-A-Lyzer^®^ G2, MWCO 3.5–5 kD) and immersed in a Falcon flask containing 10 mL of PBS (10 mM, pH 7.4) at 37 °C to simulate the physiological environment. The entire assemblies were kept under agitation (50 oscillations/min) using a Unitronic Vaivén (J.P. Selecta S.A., Barcelona, Spain) shaking water bath. At predetermined time intervals, samples (1 mL) of the release medium were withdrawn and replaced by an equal volume of pre-heated fresh release medium to maintain sink conditions. The samples were analyzed by UV-Vis spectrophotometry (Agilent 8453, Waldbronn, Germany) (λ 485 nm) and the concentration of released drug was determined using a standard calibration curve in PBS (r^2^ = 0.991).

Tests were also performed adding GSH 50 mM to the vesicle suspension in the dialysis tube to simulate the reducing environment in the tumor cells. All the release experiments were conducted in triplicate under dark conditions.

#### 2.2.6. Statistical Analysis

The results were expressed as mean ± standard deviation (SD). IBM^®^ SPSS^®^ Statistics version 26 software (Armonk, NY, USA) was used for statistical analysis of the data. One-way analysis of variance (ANOVA) with a Bonferroni multiple comparison test was used to determine statistical significance between the groups. Differences with a *p* value < 0.05 were considered statistically significant.

## 3. Results and Discussion

### 3.1. Preparation and Characterization of Blank Polymersomes

Polymersomes from the triblock copolymer mPEG–PDH–mPEG were obtained by the solvent-exchange method through the slow addition of PBS (10 mM, pH 7.4) to the polymer solution in THF. The solvent-exchange method was chosen because it is a widely applicable preparation technique due to its simplicity, easy scale-up and immediate formation of polymersomes with controlled size and narrow size distribution [27,29,34,35].

The weight fraction of the hydrophilic blocks of the amphiphilic copolymer (∫ value) played a critical role in polymersome formation [26,27,36]. The triblock copolymer mPEG–PDH–mPEG had a ∫ value of ~15% (*w*/*w*), which agreed with the ∫_PEG_ ≤ 35 ± 10% (*w*/*w*) values found for vesicular structures [5,16,27,29,37,38].

**Figure 3:**
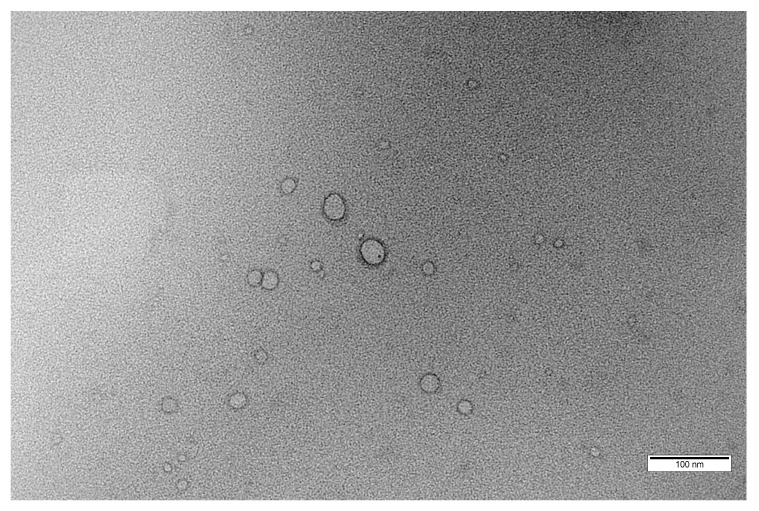
Conventional TEM image of the blank polymersomes formed at pH 7.4 with a polymer concentration of 5 mg·mL^−1^ (scale bar = 100 nm).

A combination of scattering (DLS) and imaging (TEM) techniques was used to assess the vesicular morphology, particle size and zeta potential of the polymersomes. The TEM image (Figure 3) shows the typical morphology of spherical vesicular structures with an inner hydrophilic core and outer hydrophobic shell, similar to other representative images of PEGylated polymersomes from the literature [16,18,39,40,41].

**Figure 4:**
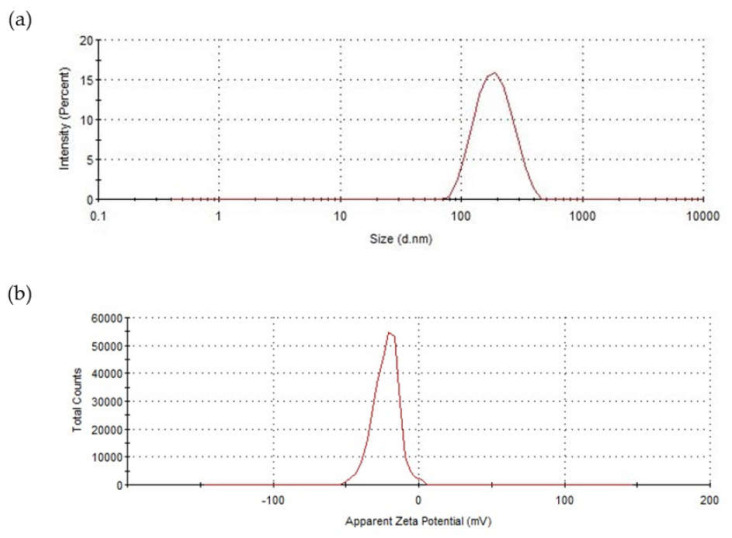
(**a**) Representative size distribution profile based on intensity and (**b**) zeta potential of blank polymersomes (measured in PBS at 25 °C).

The DLS results shown in Figure 4a revealed that the average hydrodynamic diameter of the blank polymersomes was 193.5 ± 2.9 nm with a polydispersity index (PDI) of 0.121 ± 0.011 (*n* = 3), indicating a narrow size distribution [3,42,43]. The particle size from DLS measurements was considerably higher than the range observed in the TEM images (less than 100 nm), which could have been due to the shrinkage of the PEG hydrophilic segment upon drying [21,41]. On the other hand, the hydrodynamic diameter was larger than the value of 130 nm reported by Benito et al. [33], which was attributed to the different dispersion media. Bartenstein et al. [44] also found larger sizes for poly(butadiene)-poly(ethylene oxide) (PBD-PEO) polymersomes dispersions in PBS compared to water, suggesting a reduction in the steric repulsion between the hydrophilic PEO segments in the presence of PBS. As a result, these blocks could be densely packed, leading to a smaller polymersome curvature.

The zeta potential for blank polymersomes (Figure 4b) was −22.4 ± 2.3 mV (*n* = 3), indicative of a stable nature, as high zeta potential values impede nanoparticle aggregation [12,41]. The net negative charge could be due to the presence of PEG and the pH of the dispersion medium [45,46].

**Figure 5:**
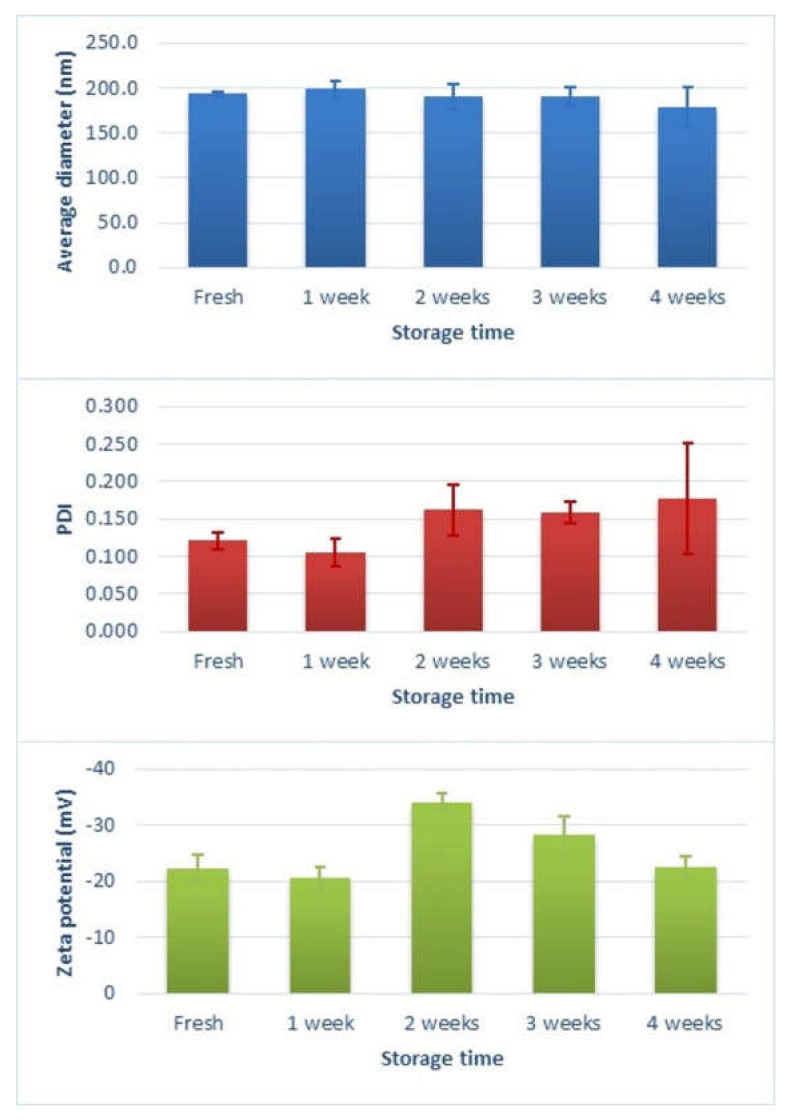
Variation in average hydrodynamic diameter, PDI and zeta potential of the blank polymersome dispersion in PBS (10 mM, pH 7.4) with time. The measurements were recorded from a fresh dispersion and after storage for four weeks at 4 °C.

Changes in particle size distribution and zeta potential were monitored each week over 1 month at a storage temperature of 4 °C to investigate the stability of the blank polymersome dispersion. The results are illustrated in Figure 5. No statistically significant changes (*p* > 0.05) in hydrodynamic diameter or size distribution were observed at 4 °C for the test period. In the second week of storage, a statistically significant increase (*p* < 0.01) in zeta potential value was noticed, indicating maximum stability, but that started to decrease significantly (*p* < 0.01) after four weeks of storage. Bartenstein et al. [44] also reported a higher stability for PBD-PEO polymersomes in PBS than in water, which was attributed to phosphate–PEO interactions.

### 3.2. Preparation and Characterization of Drug-Loaded Polymersomes

Dox·HCl loading was performed at theoretical 5 and 10 wt.% feed-weight ratios by the solvent-exchange method at pH 7.4. To improve the encapsulation efficiency of Dox·HCl, drug loading was simultaneously accomplished to the self-assembly polymeric process of mPEG–PDH–mPEG [9,35].

**Figure 6:**
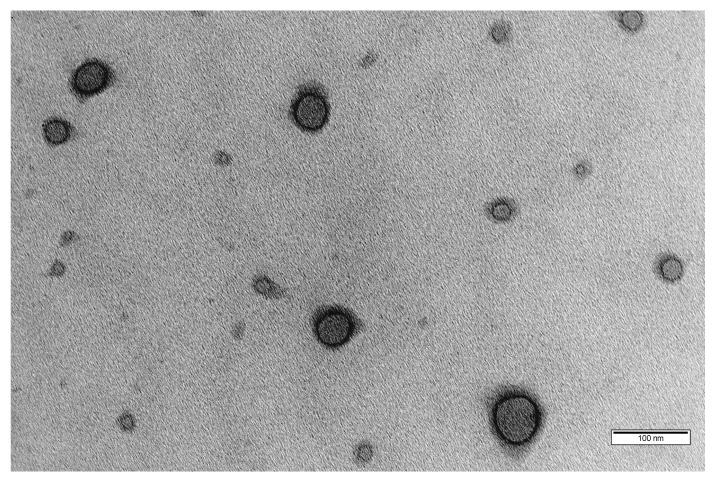
Unstained TEM image of drug-loaded (5 wt.% Dox·HCl) polymersomes (scale bar = 100 nm).

**Figure 7:**
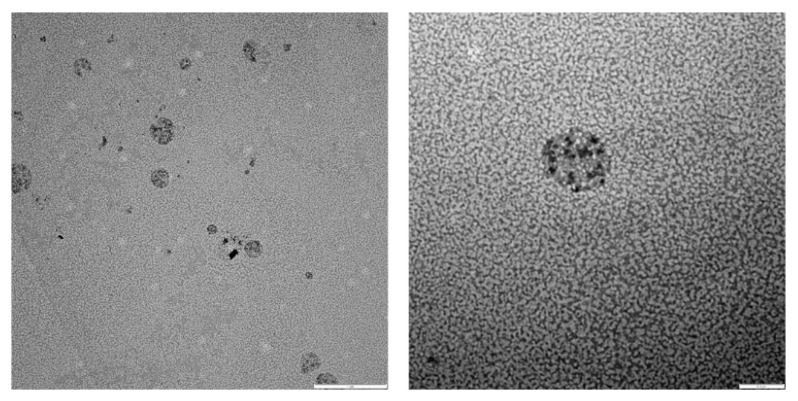
TEM images of drug-loaded (10 wt.% Dox·HCl) polymersomes stained with 1.0 wt.% phosphotungstic acid solution (scale bar = left 1 µm, right 0.2 µm).

**Figure 8:**
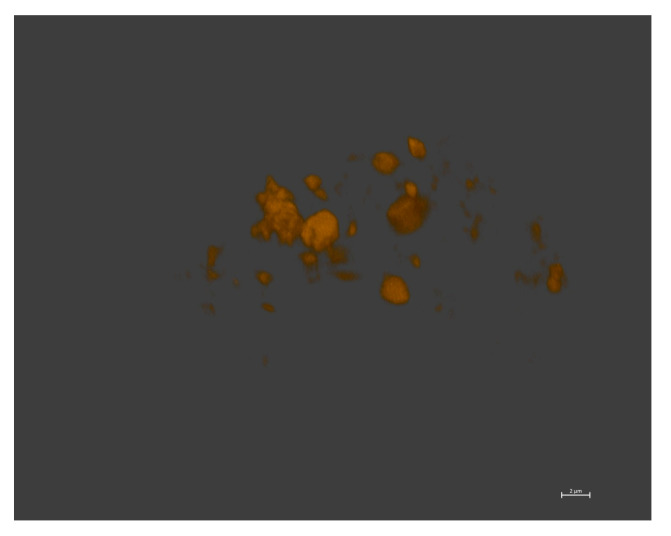
CLSM image of drug-loaded (10 wt.% Dox·HCl) polymersomes (scale bar = 2 µm).

The morphology of the self-assembled structures and the drug distribution were observed by complementary microscopic techniques. Conventional TEM images of drug-loaded (5 wt.% Dox·HCl) polymersomes (Figure 6) confirmed the spherical vesicular morphology. TEM images obtained from negatively stained drug-loaded (10 wt.% Dox·HCl) polymersomes (Figure 7) clearly showed the presence of Dox·HCl in the hydrophilic core of the vesicles [18,27,47]. Confocal laser scanning microscopy (CLSM) further aided in observing the drug distribution within the drug-loaded (10 wt.% Dox·HCl) polymersomes (Figure 8). Fluorescent polymersomes could be seen at the emission wavelength of 596 nm.

Results from the physical characterization of the drug-loaded polymersomes are illustrated in Table 1. The hydrodynamic size was significantly smaller (*p* < 0.01) than that of the blank polymersomes, which may have been due to the lower polymer volume fraction and the changes in the aggregation behavior of drug-loaded polymersomes during the loading of Dox·HCl [21,48]. The Dox·HCl feeding ratio did not significantly (*p* > 0.05) affect the average particle size or the narrow size distribution of the polymer vesicles. The particle dimensions kept then within the precept that particles smaller than 200 nm reach tumor site through EPR [12,26].

The absolute value of the zeta potential for drug-loaded (5 wt.% Dox·HCl) polymersomes (Table 1) decreased significantly (*p* < 0.01) compared to the blank polymersomes. This could have been due to partial neutralization of the negative charge of the vehicle by the positive charge of the drug that could be partially adsorbed on the polymersome surface. It was reported [1,2,4,5,38,48] that, when loaded at pH 7.4, most of the Dox molecules were protonated and might have been localized in the hydrophilic reservoir of the vesicles although a small amount of Dox could be partially adsorbed at the outer surface of the assemblies. As the zeta potential for drug-loaded (10 wt.% Dox·HCl) polymersomes significantly (*p* < 0.01) increased to values similar to those of the blank polymersomes, we postulated that the drug was preferentially localized in the inner core, as could be seen in the stained-TEM image (Figure 7). Moreover, the highly negatively charged drug vehicles were more stable and exhibited prolonged blood circulation [4,49].

### 3.3. Calculation of Drug Encapsulation Efficiency and Drug Loading Content

Encapsulation efficiency is one of the most important parameters for evaluating the quality of nanoparticulate formulations. Dox·HCl was used as a model hydrophilic anticancer drug to investigate the drug-loading capability of the designed polymersomes.

**Figure 9:**
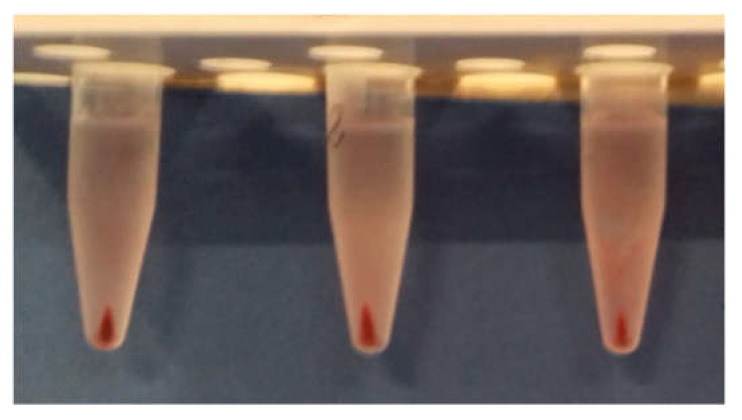
Photograph of centrifuged samples (10 wt.% Dox·HCl polymersomes dispersion).

Drug encapsulation efficiency (DEE, wt.%) and drug loading content (DLC, wt.%) were indirectly determined by HPLC after sample centrifugation. A reddish precipitate and nearly colorless supernatant were observed (Figure 9), suggesting the encapsulation of Dox·HCl into polymersomes. Results in Table 1 showed that Dox·HCl was successfully encapsulated with high drug encapsulation efficiency (>90%). Efficiency and drug-loading increased with a higher feed–weight ratio as more molecules were available for entrapment, in agreement with other authors [2,49].

### 3.4. In Vitro Drug Release Study

In vitro drug release testing is important for quality control as well as for prediction of the in vivo performance of drug delivery systems. Due to the higher DLC (9.8 wt.%) and improved stability (high negative surface charge) of 10 wt.% Dox·HCl polymersomes, this formulation was chosen for subsequent release experiments.

Drug release studies were carried out by dialysis, one of the most widely used methods for release testing of nanoparticulate systems [50,51]. For comparative purposes, the drug release behavior of drug-loaded polymersomes was assessed at pH 7.4 in the presence and absence of 50 mM GSH to mimic intracellular and extracellular redox environments, respectively. The tripeptide glutathione was chosen as the reducing agent to evaluate the redox responsive nature of polymersomes due to the higher GSH concentration of cancer cells compared to healthy cells [16,27,28,29]. The concentration of GSH was selected based on the results obtained by Anajafi et al. [27,47] and Karandish et al. [28], who reported deformation or disruption of the vesicles when incubated with 50 mM GSH.

**Figure 10:**
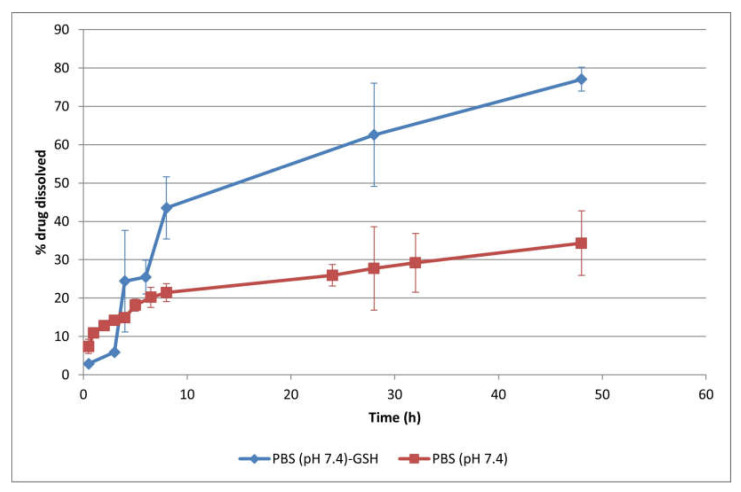
Cumulative in vitro release profiles of drug-loaded (10 wt.% Dox·HCl) polymersomes in PBS (10 mM, pH 7.4) with or without 50 mM GSH at 37 °C (mean ± SD, *n* = 3).

The cumulative release profiles of drug-loaded (10 wt.% Dox·HCl) polymersomes in PBS (10 mM, pH 7.4) are illustrated in Figure 10. They exhibited a prolonged drug release behaviour, with 34.3 ± 8.4% drug released at physiological pH after 48 h. This cumulative release percentage was in the range obtained for other PEGylated polymersomes when tested at pH 7.4 for 24–48 h [18,37,40,49]. The prolonged drug release at physiological conditions was attributed to the structural stability of the polymersomes, so drug diffusion through their membrane was postulated as the main release mechanism under these conditions.

The drug release rate increased in the presence of 50 mM GSH (Figure 10), with a cumulative drug release up to 77.1 ± 3.1% after 48 h. The disulfide linkages in the hydrophobic block of the copolymer were cleaved in the reductive environment, leading to the rupture of polymersomes, which subsequently accelerated drug release. The chemical degradation of the PDH block under physiological conditions and in the presence of GSH was demonstrated in previous studies by a combination of GPC, AFM and SEM techniques [33,52,53]. The degradation profile was characterized by a rapid decrease in molecular weight during the first two days, followed by a second degradation step at a low rate. As a consequence, the spherical shape of the polymersomes was destroyed. Other authors also found an abrupt drug release of doxorubicin from redox-responsive polymersomes in the presence of high concentrations of GSH (10–50 mM) [14,16,47,54].

Based on these results, a low doxorubicin release was predicted at normal physiological conditions as well as faster drug release in the redox environment of cancer cells after polymersomes uptake. Together with the high concentration of GSH, the mildly acidic endolysosomal pH also contributed to accelerated drug release through the protonation of the doxorubicin glycosidic amine group (pK_a_ 8.25) and the consequent increase in its hydrophilicity and solubility [14,16].

The drug release kinetics from the designed polymersomes were also investigated. Mathematical modeling of drug release profiles is essential for elucidating drug release mechanisms and providing valuable information for formulation optimization to achieve the desired release rate [50,51]. Because drug release from nanoparticulate systems is a complex process where several mechanisms may be involved, different kinetic release models were applied such as zero-order (3), first-order (4), Higuchi (5), Korsmeyer–Peppas (6), Peppas–Sahlin (7), Hixon-Crowell (8) and Baker-Lonsdale (9) [55,56].

(3) $$\frac{M_{t}}{M_{\infty }}=k_{0}\cdot t$$

(4) $$\ln\left(1-\frac{M_{t}}{M_{\infty }}\right)=-k_{1}\cdot t$$

(5) $$\frac{M_{t}}{M_{\infty }}=k_{H}\cdot t^{0.5}$$

(6) $$\frac{M_{t}}{M_{\infty }}=k_{K}\cdot t^{n}\;\;$$

(7) $$\frac{M_{t}}{M_{\infty }}=k_{d}\cdot t^{m}+k_{r}\cdot t^{2m}$$

(8) $${\left(1-\frac{M_{t}}{M_{\infty }}\right)}^{1/3}=-k_{HC}\cdot t$$

(9) $$\frac{3}{2}\left[1-{\left(1-\frac{M_{t}}{M_{\infty }}\right)}^{2/3}\right]-\frac{M_{t}}{M_{\infty }}=k_{BL}\cdot t$$

where *M_t_/M_∞_* is the fractional drug release at time *t (M_∞_* is considered equivalent to the drug loading); *k*_0_ is the zero-order release rate constant; *k_1_* is the first-order release rate constant; *k_H_* is the Higuchi kinetic constant; *k_K_* is the Korsmeyer kinetic constant; *n* is a release exponent that depends on the release mechanism and the geometry of the system; *k_d_* and *k_r_* are the diffusion and relaxation rate constants, respectively; *m* is the purely Fickian diffusion exponent for a device of any geometrical shape which exhibits controlled release; *k_HC_* is the Hixon–Crowell dissolution rate constant and *k_BL_* is the Baker–Lonsdale release rate constant.

The optimum values for the parameters in each equation (Table 2) were determined by linear or non-linear least-squares fitting methods using SPSS^®^ Statistics 26 software. The adjusted coefficient of determination (*r*^2^*_adj_*) and F-ratio probability were used to test the applicability of the release models.

The drug release profile at pH 7.4 best fitted the Korsmeyer–Peppas, Peppas–Sahlin and Baker-Lonsdale models. In the case of pure Fickian release from a spherical geometry, the exponent *n* in the Korsmeyer–Peppas equation had the limiting value of 0.43, while the value for Case II transport (relaxation or erosion) was *n* = 0.85. The low *n* value (0.31) in Table 2 could be indicative of a quasi-Fickian diffusion. Similar values were found by other authors for doxorubicin release from polymersomes in pH 7.4 dissolution media [2,49]. Sanson et al. [2] attributed this deviation from a pure diffusion release mechanism to the aggregation of Dox in the membrane of the polymersomes. Hence, drug solubilization was necessary before molecular diffusion. Additionally, the prevalence of *k_d_* over *k_r_* in the Peppas–Sahlin model revealed that diffusion predominated over relaxation or erosion. The accurate fit of the release profile to the Baker–Lonsdale model also confirmed that drug release from the spherical matrix predominantly followed a diffusion-controlled process.

In general, the drug release profile at pH 7.4 in the presence of 50 mM GSH showed higher variability and poorer correlation to the fitting models than in the absence of the reduction agent due to the rupture/degradation of the polymersomes. The *n* value in the Korsmeyer–Peppas equation increased to 0.76, indicating an anomalous transport. This could have been attributed to the breakdown of disulfide bonds in the polymersomes that accelerated drug release by a combined mechanism of diffusion of the drug molecules through the membrane and erosion of the polymeric membrane. The increase in *k_HC_* from the Hixon–Crowell cube root law also indicated a change in the surface area and diameter of the polymer systems due to erosion. Simultaneously, faster diffusion rate constants were observed in the Higuchi, Peppas–Sahlin and Baker–Lonsdale models due to the loss of membrane integrity. An increase in the contribution of the erosion mechanism in the presence of different concentrations of GSH has been reported for other types of biodegradable redox-responsive polymersomes [14].

## 4. Conclusions

We successfully obtained polymersomes from the self-assembly of the amphiphilic biodegradable mPEG–PDH–mPEG triblock copolymer in a phosphate buffer solution (pH 7.4, 10 mM) at room temperature. By means of the proposed optimized self-assembly method, the anticancer drug Dox·HCl was encapsulated into the polymersomes’ hydrophilic core (10 wt.% feeding ratio) with a remarkably high loading efficiency (98% wt.%). The size of the resulting polymersomes (124 nm) was also appropriate for achieving passive targeting via the EPR effect, and the negative zeta potential (−24 mV) confirmed their stability. These drug-loaded polymersomes demonstrated prolonged drug release under physiological conditions mainly governed by drug diffusion through the polymeric membrane. Furthermore, the redox-sensitive nature of the polymersomes accelerated the release of doxorubicin under reductive conditions (50 mM GSH) by a combined mechanism of diffusion and erosion. In conclusion, the designed polymersomes represented a versatile smart platform that combined the advantages of PEGylated formulations that prolong circulation half-life with the benefit of a specific activation in a reductive environment to trigger the rapid release of doxorubicin at the tumor site. Therefore, this study provided proof that the obtained redox-responsive polymersomes could be a potential candidate for further exploration into controlled drug delivery applications.

## Figures and Tables

**Table 1:** Physicochemical characteristics of the drug-loaded polymersomes.

Dox·HCl (Theoretical Load)	Average Diameter (nm)	PDI	Zeta Potential (mV)	DEE (wt.%)	DLC (wt.%)
5 wt.%	139.1 ± 7.5	0.151 ± 0.016	−7.0 ± 1.5	93.83 ± 0.01	4.69 ± 0.00
10 wt.%	124.2 ± 12.0	0.164 ± 0.071	−23.8 ± 0.9	98.26 ± 0.10	9.83 ± 0.01

Data represent mean ± SD, *n* = 3.

**Table 2:** Mathematical modeling and drug release kinetics from drug-loaded (10 wt.% Dox·HCl) polymersomes.

Kinetic Model	Parameters	pH 7.4 Release Medium	pH 7.4/50 mM GSH Release Medium
Zero-order (3)	*k*_0_ (h^−1^) *r*^2^*_adj_*	0.0049 0.8617 (*F* = 70)	0.0146 0.8074 (*F* = 26)
First-order (4)	*k*_1_ (h^−1^) *r*^2^*_adj_*	0.0065 0.8977 (*F* = 98)	0.0297 0.9348 (*F* = 87)
Higuchi (5)	*k_H_* (h^−0.5^) *r*^2^*_adj_*	0.0392 0.9572 (*F* = 247)	0.1230 0.9154 (*F* = 66)
Korsmeyer–Peppas (6)	*n* *k_K_* (h^−*n*^) *r*^2^*_adj_*	0.31 1.1077 0.9762 (*F* = 452)	0.76 1.0549 0.8466 (*F* = 34)
Peppas–Sahlin (7)	*k_d_* (h^−0.43^) *k_r_* (h^−0.86^) *r*^2^*_adj_*	0.0827 −0.0048 0.9755 (*F* = 220)	0.2489 −0.0125 0.9174 (*F* = 34)
Hixson–Crowell (8)	*k_HC_* (h^−1^) *r*^2^*_adj_*	0.0020 0.8862 (*F* = 87)	0.0077 0.8993 (*F* = 55)
Baker–Lonsdale (9)	*k_BL_* (h^−1^) *r*^2^*_adj_*	0.0005 0.9700 (*F* = 356)	0.0036 0.9829 (*F* = 347)

*k*_0_, zero-order release rate constant; *k*_1_, first-order release rate constant; *k_H_*, Higuchi kinetic constant; *n*, release exponent; *k_K_*, Korsmeyer kinetic constant; *k_d_*, diffusion kinetic constant; *k_r_*, relaxation kinetic constant; *k_HC_*, Hixon–Crowell dissolution rate constant; *k_BL_*, Baker–Lonsdale release rate constant; *r*^2^*_adj_*, adjusted coefficient of determination; *F*, *F* distribution for residual variance analysis (*p* < 0.01).

## Data Availability

Data are contained within the article.
